# Enhanced Antifungal Efficacy of Validamycin A Co-Administered with *Bacillus velezensis* TCS001 against *Camellia anthracnose*

**DOI:** 10.3390/plants13192743

**Published:** 2024-09-30

**Authors:** Zhilei Chen, Hao Cao, Jing Jin, Zhong Li, Shouke Zhang, Jie Chen

**Affiliations:** 1 Zhejiang Green Pesticide 2011 Collaborative Innovation Center, Zhejiang Agriculture and Forestry University, Hangzhou 311300, China; chenluocen@foxmail.com (Z.C.); ch990805hao@163.com (H.C.); jinjing2022@zafu.edu.cn (J.J.); 2 Zhejiang Tonglu Huifeng Biosciences Co., Ltd., Hangzhou 311500, China; lizhong245@sina.cn

**Keywords:** validamycin A, *Bacillus velezensis*, antifungal activity, *Camellia oleifera*, plant immunity

## Abstract

Anthracnose, a fungal disease harming fruit trees and crops, poses a threat to agriculture. Traditional chemical pesticides face issues like environmental pollution and resistance. A strategy combining low-toxicity chemicals with biopesticides is proposed to enhance disease control while reducing chemical use. Our study found that mixing validamycin A (VMA) and *Bacillus velezensis* TCS001 effectively controlled anthracnose in *Camellia oleifera*. The combination increased antifungal efficacy by 65.62% over VMA alone and 18.83% over TCS001 alone. It caused pathogen deformities and loss of pathogenicity. Transcriptomic analysis revealed that the mix affected the pathogen’s metabolism and redox processes, particularly impacting cellular membrane functions and inducing apoptosis via glycolysis/gluconeogenesis. In vivo tests showed the treatment activated *C. oleifera*’s disease resistance, with a 161.72% increase in polyphenol oxidase concentration in treated plants. This research offers insights into VMA and TCS001’s mechanisms against anthracnose, supporting sustainable forestry and national edible oil security.

## 1. Introduction

In light of the growing intricacy of the global grain and oil trade, China, the world’s largest consumer of edible oils, is obliged to expand the cultivation area of oil-bearing crops in order to guarantee the production of edible oil domestically [1,2]. *Camellia oleifera* is widely acknowledged as the most suitable woody oil crop owing to its superior oil quality and high economic value [3,4]. The oil derived from *C. oleifera* has high concentrations of monounsaturated fatty acids [5], particularly oleic acid, which constitutes ~80% of the total oil content [6]. Additionally, *C. oleifera* oil contains distinctive chemical components and possesses substantial health-promoting properties [7]. Nevertheless, as the cultivation area of *C. oleifera* expands, the issue of disease in this species, particularly the anthracnose disease, becomes increasingly prevalent [8]. This disease is predominantly caused by the pathogen of genus *Colletotrichum* spp. [9]. In extreme cases, the anthracnose disease of *C. oleifera* can result in the loss of flowers and fruits, the death of branches, and ultimately, the death of the root system of the entire plant. This leads to a significant reduction in yield, which could range from 20% to 40%, and even up to 80% in severe cases. This loss in production can cause substantial economic losses to local forestry and *C. oleifera* enterprises [10,11].

At present, the control of anthracnose disease in *C. oleifera* is predominantly reliant on chemical methods [12,13]. However, given that *C. oleifera* is predominantly cultivated in mountainous regions and areas designated for the protection of water sources [14,15], the prolonged utilization of chemical pesticides may result in the contamination of water sources and an increased concentration of pesticide residues in the oil product [16,17]. Furthermore, anthracnose in *C. oleifera* has demonstrated a notable degree of pesticide resistance [18]. Therefore, it is becoming increasingly important to seek out healthier and more environmentally friendly methods for the control of plant diseases [19]. The biological control method, an emerging approach to disease control, has gradually gained attention [20,21]. The utilization of *Bacillus* species in microbial control has become a prominent area of research owing to environmental friendliness, reduced likelihood of developing drug resistance, safety for humans and animals, ease of access, and the strong sustainability prospects of this method. *Bacillus* species effectively inhibit the pathogen responsible for anthracnose and activate the immune system of the plant by inducing systemic acquired resistance [22,23]. Numerous species of *Bacillus* exist; examples include *Bacillus subtilis*, *B. amyloliquefaciens*, *B. polymyxa*, and *B. velezensis*. The *B. velezensis* TCS001 strain used in this investigation was obtained from marine mud in the Bohai Sea off the coast of China. The strain has undergone stages of genetic modification, stabilization, identification, and nomenclature. Previous studies have demonstrated that this strain exhibits a pronounced inhibitory effect against a variety of fungi causing plant diseases and promotes plant growth by inducing stress resistance [24]. Biological plant disease resistance activators, such as the protein types, oligosaccharides, and glycoprotein types, have demonstrated efficacy in the control of plant diseases [25]. Validamycin A (VMA), also known as *Jinggangmycin* in China, is an amino-oligosaccharide antibiotic produced by *Streptomyces hygroscopicus*. It induces host resistance reactions in tomatoes and rice and has been widely used in many countries to control crop diseases caused by *Rhizoctonia* species [26]. Our laboratory discovered (dates not published) that VMA can also stimulate the intrinsic immune response of plants. Therefore, this study explored the disease resistance-activating function of VMA in *C. oleifera* and its pathogen, with a focus on its potential mechanisms.

The objective of this research on the combination of biological control microorganisms and chemical pesticides is to address the environmental pollution, potential health risks to humans, and poor sustainability associated with chemical pesticides [27]. The goal is to reduce pesticide application while improving control effectiveness. The purpose of this study is to explore whether the combined use of the biological disease control bacterium TCS001 and the biological disease resistance activator VMA can activate disease resistance in *C. oleifera* plants and to conduct internal mechanism research, providing an effective solution to the problems caused using chemical pesticides. This approach aims to facilitate the implementation of an integrated chemical and biological control method in sustainable forestry, ensuring the security of the national edible oil supply.

## 2. Results

### 2.1. In Vitro Inhibitory Activity and Affinity of Colletotrichum siamense

**Figure 1:**
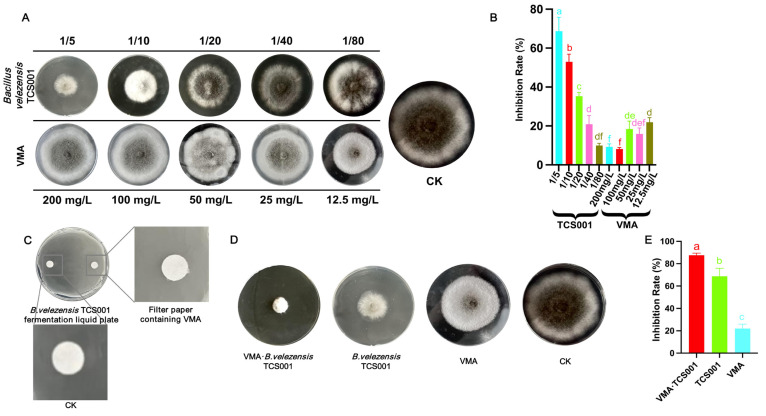
Results of the in vitro assay for the inhibitory activity against *Colletotrichum siamense* and evaluation of affinity. (**A**) The in vitro antifungal effects of TCS001 fermentation filtrate at serial dilutions of 1/5, 1/10, 1/20, 1/40, and 1/80 (fermentation filtrate to medium) and validamycin A at concentrations of 200, 100, 50, 25, and 12.5 mg/L. (**B**) The in vitro inhibition rates for Petri dishes containing TCS001 fermentation filtrate at the aforementioned dilutions. This panel also shows the in vitro inhibition rates for Petri dishes treated with VMA at concentrations of 200, 100, 50, 25, and 12.5 mg/L. Different lowercase letters in the figure indicate a significant difference at *p <* 0.05 level as determined using a Duncan’s new multiple range test. (**C**) Solid LB agar plates inoculated with the TCS001 fermentation broth, with filter paper disks soaked in VMA and sterile water applied on top. (**D**) Plates after screening the optimal inhibitory concentration of VMA · *B. velezensis* TCS001 combined treatments, where both agents are mixed in a 1:1 volumetric ratio (VMA at 12.5 mg/L, TCS001 at a fermentation filtrate to medium ratio of 1/5), along with separate treatments of TCS001 and VMA, with CK as the control group. (**E**) shows the inhibition rates following the application of VMA at 12.5 mg/L and TCS001 at a fermentation filtrate to medium ratio of 1/5, as well as the combined VMA and TCS001 treatment. Different lowercase letters in the figure indicate a significant difference at *p <* 0.05 level as determined using a Duncan’s new multiple range test.

The antimicrobial activity of various concentrations of VMA and the fermentation filtrate of TCS001 against *Co. siamense* was evaluated (Figure 1A). According to the inhibition rates (Figure 1B), the most effective treatment with VMA was at a concentration of 12.5 mg/L, which achieved an inhibition rate of 21.92%. Moreover, the optimal condition for the treatment group comprising the fermentation filtrate of TCS001 was a liquid-to-water ratio of 1:5, which resulted in an inhibition rate of 68.71% (Figure 1B). The compatibility test results (Figure 1C) demonstrated that no inhibition zones were formed when both agents were combined. This indicated that VMA and TCS001 were compatible and could be further tested in combination. After completing the aforementioned steps, a mixture (1:1 *v*/*v*) consisting of VMA (12.5 mg/L) and TCS001 fermentation filtrate (liquid-to-water ratio of 1:5) was prepared. Following the aforementioned procedures, the same treatments used in the in vitro plate assays were applied, and the antimicrobial effects of the mixture were evaluated using identical investigation and data analysis methods over the same time period (Figure 1D,E). The combined treatment resulted in an inhibition rate of 87.54% (Figure 1E), which represented a significant enhancement in antimicrobial efficacy compared with the separate treatments of VMA and TCS001, with improvements of 65.62% and 18.83%, respectively. This finding indicated that the combined application of VMA and TCS001 had a synergistic inhibitory effect on the growth of *Co. siamense*. This finding provided direct evidence of the feasibility of the co-application of chemical and biological pesticides.

### 2.2. Three-Dimensional Spatial Structure and Pathogenicity of Co. siamense Mycelium under Different Treatments

**Figure 2:**
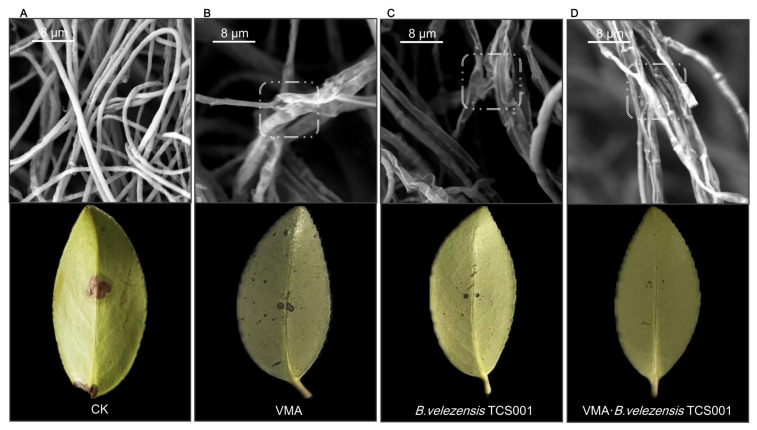
Relationship between morphological variations of *Colletotrichum siamense* and pathogenicity (Each panel is divided into two parts: the upper part shows the growth of hyphae observed under SEM, and the lower part represents the pathogenicity of the hyphae under that condition). (**A**) Control treatment with sterile water. (**B**) Fermentation filtrate of *Bacillus velezensis* TCS001 at a dilution of 1/5 (liquid to water). (**C**) Mixture of 12.5 mg/L VMA and the fermentation filtrate of *B. velezensis* TCS001 at a dilution of 1/5 (liquid to water). (**D**) Mycelium (upper images) and leaf disease conditions (lower images) observed via SEM following treatment with 12.5 mg/L of validamycin A.

The spatial structure of the *Co. siamense* mycelium and its relationship with pathogenicity were investigated via scanning electron microscopy (SEM). In the control treatment with deionized water (Figure 2A), the mycelium exhibited a plump morphology with an average lesion diameter of 8.3 mm. The three different treatments all resulted in similar morphological abnormalities in the fungi, characterized by mycelial breakage, twisting, and deformation, along with a decrease in pathogenicity. Notably, despite the relatively low antifungal rate observed in the culture dish of the VMA treatment group, the mycelium exhibited deformations such as twisting and damage (Figure 2B). The TCS001 treatment group exhibited a greater number of representative morphological abnormalities (shrinkage and twisting) in the mycelium (Figure 2C). In the mixed treatment group (Figure 2D), the morphological abnormalities (breakage and twisting) exhibited characteristics of both VMA and TCS001 and deformation was more pronounced. The findings of this study demonstrated that the three types of agents affected *Co. siamense* to varying degrees during the infection process of anthracnose and resulted in significant changes in the pathogenicity of the fungus. Notably, the fungus completely lost its pathogenicity under the combined treatment of VMA and TCS001, which effectively mitigated the occurrence of the plant disease.

### 2.3. Assembly and Analysis of the Transcriptome of Co. siamense with Different Pathogenicity

This section describes the assembly and identification of the transcriptome of *Co. siamense* with varying pathogenic capacities. Transcriptome analysis was conducted on sixteen samples from four treatment groups. The analysis provided a substantial amount of data with a total of 101.86 GB of clean data generated. Each sample exceeded 25 GB with a Q30 base percentage above 95.88%. The statistical results of the transcriptome data for the 16 samples are presented in Table S1. The raw reads ranged from 37,153,280 to 52,880,670. After quality assessment and filtering of low-quality reads, the number of clean reads ranged from 36,867,896 to 52,301,274. After the alignment of the clean reads with the specified reference genome, the number of aligned reads ranged from 31,991,954 to 46,421,164 with an alignment rate between 83.79% and 88.76%. The Q30% values ranged from 95.88% to 96.91%, which indicated that the transcriptome sequencing results were of high accuracy and that the data obtained were suitable for subsequent analysis.

### 2.4. Analysis of Transcriptome Data Using Weighted Gene Co-Expression Network Analysis (WGCNA)

**Figure 3:**
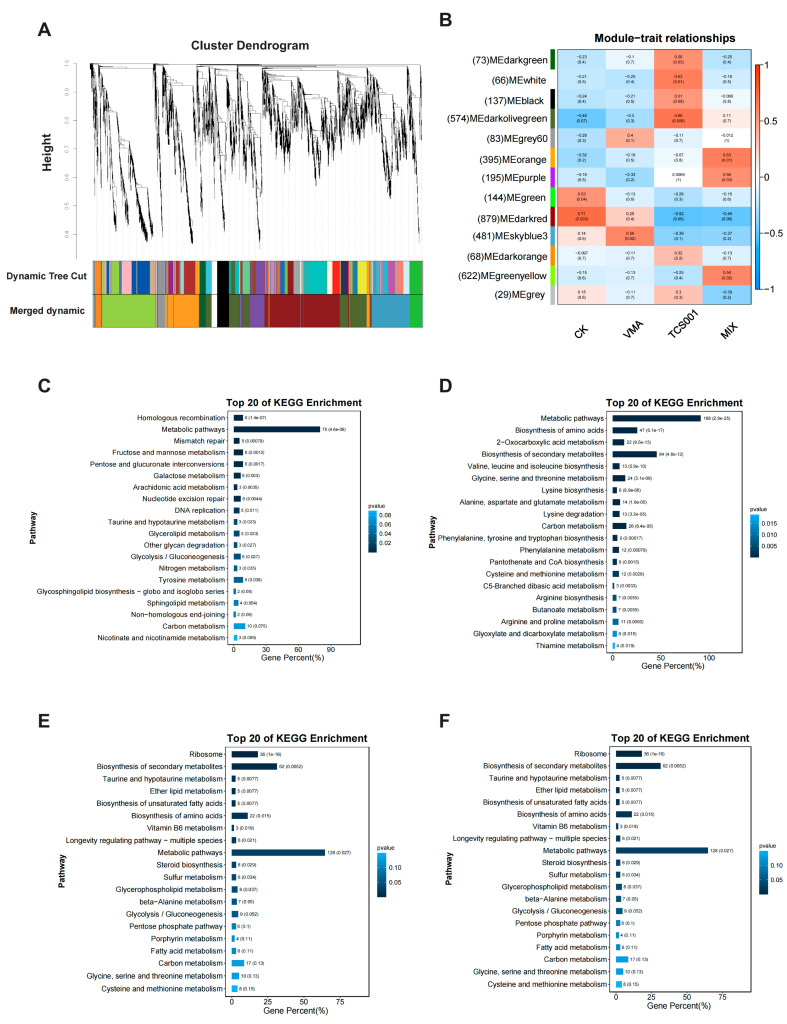
WGCNA was utilized to examine the comprehensive gene expression data derived from the transcriptome. (**A**) Unigene dendrogram derived from transcriptome data, presented in a color-coded format to facilitate the visualization of co-expressed genes with distinctive patterns. The “Dynamic Tree Cut” and “Merged Dynamic” labels indicate the optimal and merged co-expression heights, respectively. (**B**) Treatment correlation heatmap for *Colletotrichum siamense* and modules, with gene counts highlighted. CK represents the sterile water control group, validamycinA is 12.5 mg/mL validamycin A, TCS001 is *Bacillus velezensis* filtrate at a 1/5 ratio, and MIX is the combination of treatments. (**C**–**F**) KEGG analysis bar charts for *Co. siamense* phenotype modules across treatments (CK, VMA, TCS001, and MIX), with the number of genes and associated *p*-values highlighted.

The WGCNA method was employed to identify modules. The optimal soft thresholding power was set to 0, and a minimum module size of 30 was used. The transcriptome data were clustered into 13 modules (based on consistent expression trends): darkgreen, white, black, darkolivegreen, gray60, orange, purple, green, darkred, skyblue3, darkorange, greenyellow, and gray (Figure 3A). Each module represents a similar expression pattern. For example, the darkred module contains the most unigenes, amounting to 879 genes, each of which has the same expression pattern, that is, there are 879 co-expression genes in this module. 

Furthermore, a phenotypic correlation analysis was conducted based on the classification of different morphological and pathogenic *Co. siamense*, to associate the 13 transcriptome-assembled modules with the phenotype of pathogenicity (Figure 3B). A *p*-value cutoff of less than 0.05 revealed a significant positive correlation between the normal growth control group (CK) phenotype and the darkred (879 unigenes) and green (144 unigenes) modules, with correlation coefficients of 0.71 and 0.53, respectively. The VMA-treated *Co. siamense* phenotype exhibited a significant positive correlation with the skyblue3 module, which comprised 461 unigenes. The TCS001-treated *Co. siamense* phenotype was significantly positively correlated with the darkolivegreen (574 unigenes), white (66 unigenes), darkgreen (73 unigenes), and black (137 unigenes) modules, with correlation coefficients of 0.68, 0.63, 0.58, and 0.51, respectively. The MIX-treated *Co. siamense* phenotype exhibited a positive correlation with the orange (395 unigenes), greenyellow (622 unigenes), and purple (195 unigenes) modules, with correlation coefficients of 0.63, 0.58, and 0.56, respectively.

The Kyoto Encyclopedia of Genes and Genomes (KEGG) enrichment analysis of the phenotype-related modules of *Co. siamense* after different treatments revealed significant metabolic pathways. In the control group (Figure 3C), the most significantly enriched pathways were metabolic pathways, fructose and mannose metabolism, and pentose and glucuronate interconversions. Notably, the metabolic pathways were markedly enriched in the citrate cycle [TCA (tricarboxylic acid cycle) cycle)] and microbial metabolism in diverse environments, with 19 and 16 unigenes, respectively. In the VMA treatment group (Figure 3D), the predominantly enriched pathways were metabolic pathways and the biosynthesis of secondary metabolites. The former was predominantly represented by glycolysis/gluconeogenesis and alanine, aspartate, and glutamate metabolism, which included 20 and 18 unigenes, respectively. In the TCS001 treatment group (Figure 3E), the predominantly enriched pathways were also metabolic pathways and the biosynthesis of secondary metabolites. The metabolic pathways were significantly enriched in the citrate cycle and in glycolysis/gluconeogenesis, with 22 and 21 unigenes, respectively. In the MIX treatment group (Figure 3F), the predominantly enriched pathways were metabolic pathways, the biosynthesis of secondary metabolites, and glycolysis/gluconeogenesis. The metabolic pathways were mostly enriched in the citrate cycle and microbial metabolism in diverse environments, with 24 and 17 unigenes involved, respectively.

By conducting a KEGG enrichment analysis on all unigenes in the significantly positively correlated *Co. siamense* phenotype-related module after different treatments, the analysis of the pathways in which the unigenes are located showed that the citrate cycle, microbial metabolism in diverse environments, and glycolysis/gluconeogenesis were the pathways repeatedly enriched in all treatments. Thus, the WGCNA indicated that VMA and TCS001 predominantly induced deformities and loss of pathogenicity by regulating genes within these three pathways.

### 2.5. Analysis of Differentially Expressed Genes in Co. siamense with Different Pathogenicity

Transcriptome analysis was conducted (with the deionized water-treated CK group as the control) to investigate the response mechanisms of *Co. siamense* to VMA (VMA group), TCS001 (TCS001 group), and the combination of VMA and TCS001 (MIX group) treatments (Table 1). The analysis revealed that, compared with the CK group, the VMA, TCS001, and MIX groups exhibited 229 (126 upregulated and 103 downregulated), 2221 (1172 upregulated and 1049 downregulated), and 2188 (1310 upregulated and 878 downregulated) differentially expressed genes (DEGs), respectively. Furthermore, this study compared the DEGs between the VMA group and the TCS001 and MIX groups. This comparison revealed 1508 (705 upregulated and 803 downregulated) DEGs between the VMA and TCS001 groups and 1412 (793 upregulated and 619 downregulated) DEGs between the VMA and MIX groups. Additionally, 484 (359 upregulated and 125 downregulated) DEGs were identified when the TCS001 group was compared with the MIX group.

**Figure 4:**
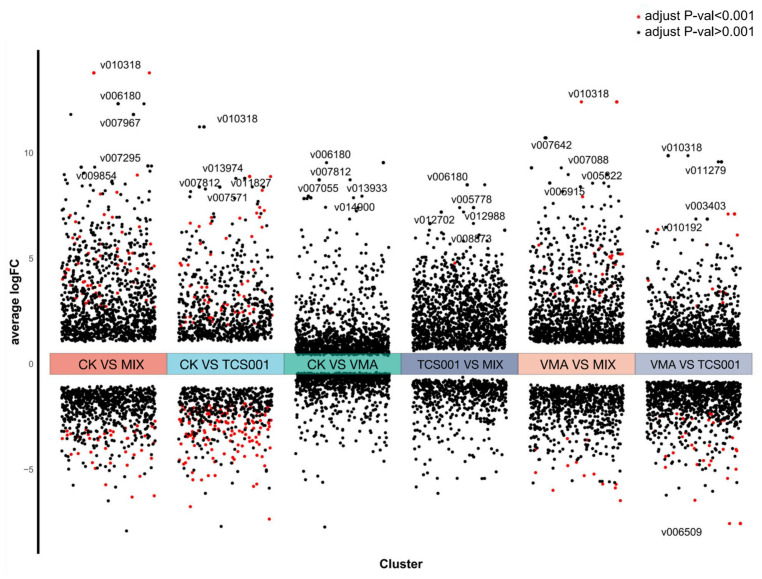
Volcano plot analysis of gene expression distribution in samples subjected to various treatment conditions.

A filter criterion of FDR < 0.05 and log2 FC ≥ 1 was applied to analyze the top 2000 DEGs based on *p*-value (Figure 4). The results demonstrated that the DEGs identified in the comparisons of CK vs. VMA and CK vs. TCS001 exhibited expression level changes between −10 and 10. In contrast, the DEG profiles from the comparison of CK vs. VMA and TCS001 (MIX group) exhibited expression level changes between −10 and 15. In pairwise comparisons of treatment groups, the DEGs from the VMA vs. TCS001 comparison exhibited expression level changes between −10 and 10, while those from VMA vs. MIX and TCS001 vs. MIX exhibited expression level changes between −10 and 15. These findings indicated that the response of *Co. siamense* to VMA and TCS001 treatments was relatively mild, with only a few genes exhibiting changes in transcription levels and low expression levels in the case of the VMA treatment. The TCS001 treatment resulted in greater changes in transcription levels compared with the VMA treatment. The combined treatment with VMA and TCS001 produced the most intense response, characterized by significant changes in the transcription levels of many genes and high expression levels. In pairwise comparisons of the treatments, the VMA and TCS001 combination demonstrated considerable alterations in transcription levels, and expression levels remained high. This indicated that the combined treatment with VMA and TCS001 had a more pronounced influence on the gene expression of *Co. siamense* than the use of either agent alone.

### 2.6. Analysis of Functional Enrichment of Anthracnose Elements in C. oleifera Treated with Different Chemicals

**Figure 5:**
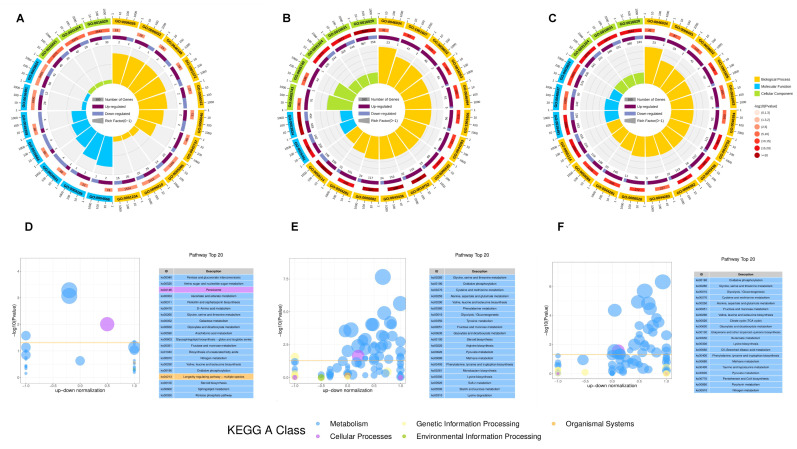
Enrichment analysis of DEGs through GO and KEGG pathways. (**A**–**C**) Circular diagrams of GO enrichment analysis for DEGs; comparisons of the CK group with the VMA group (**A**), the CK group with the TCS001 group (**B**), and the CK group with the VMA andTCS001 combined group. (**D**–**F**) The z-score bubble plots of the top 20 significantly enriched differential pathways in the CK group/VMA group, CK group/TCS001 group, and CK group/VMA and TCS001 group. In the combined chart, the horizontal axis of the bubble plot is the up/down normalization coefficient, the vertical axis is −log10*p*value, and different colors represent different functional classifications. The orange threshold line is *p* value = 0.05; the size of the bubble represents the number of genes enriched in the current term (pathway) (that is, Up + Down); while the table on the right is the 20 GO terms with the smallest *p* value.

To evaluate the effect of different treatments on the biological activity of *Co. siamense*, we conducted Gene Ontology (GO) and KEGG enrichment analyses on all DEGs (Figure 5). In the GO enrichment analysis, the top 20 enriched GO terms were selected for each treatment through comparisons of the CK group with the VMA group, the TCS001 group, and the VMA and TCS001 combination (Figure 5A–C). In the diagram, the first circle represents the enriched categories, with the outer circle serving as a scale for the number of genes. Different colors indicate different categories; the second circle shows the number of genes in the background for that category, as well as the Q-value or *p*-value. The longer the bar, the more genes there are, and the smaller the value, the redder the color; the third circle is a bar chart showing the ratio of up- and down-regulated genes, with dark purple representing the proportion of up-regulated genes and light purple representing the proportion of down-regulated genes; specific values are displayed below; when the input number of differential genes is only one column (without distinguishing between up- and down-regulation), the third circle shows the total number of foreground genes; the fourth circle displays the RichFactor values for each category (the number of foreground genes in that category divided by the number of background genes), with each small grid in the background auxiliary line representing 0.1. In the category of biological processes, transmembrane transport (GO:0055085) was significantly enriched in both the CK vs. VMA and CK vs. VMA and TCS001 groups. Additionally, a series of GO terms related to metabolic processes were highly enriched in both the CK vs. TCS001 and CK vs. VMA and TCS001 groups, which indicated a high degree of similarity in biological processes between these two groups. These GO terms encompassed a range of processes, which included oxoacid metabolic processes, organic acid metabolic processes, carboxylic acid metabolic processes, and oxidation–reduction processes. Furthermore, the unique GO:0006091 term in the CK vs. VMA and TCS001 group, annotated as the generation of precursor metabolites and energy, may be associated with the high antifungal rate and loss of pathogenicity observed following the combination treatment.

In the category of molecular functions, all three comparison groups exhibited common enrichment in catalytic activity (GO:0003824) and oxidoreductase activity (GO:0016491). In the category of cellular components, all groups exhibited enrichment in the integral component of membrane (GO:0016021), membrane (GO:0016020), and intrinsic component of membrane (GO:0031224).

In the KEGG enrichment analysis composite graph (Figure 5D–F), the horizontal axis of the bubble chart represents the up/down normalization coefficient, and the vertical axis represents the negative logarithm of the *p*-value (−log10*p*value). Different colors indicate different functional categories, with the orange threshold line representing a *p*-value of 0.05. The size of the bubbles indicates the number of genes enriched in the current term (pathway), i.e., the sum of up- and down-regulated genes. The table on the right lists the top 20 GO terms with the smallest *p*-values. This chart provides a visual representation of the significantly enriched functions within different functional categories. The KEGG enrichment analysis revealed that the CK vs. VMA group had the highest number of genes annotated in pathways such as pentose and glucuronate interconversions, amino sugar and nucleotide sugar metabolism, and peroxisomes. Notably, the annotated genes in the other two comparison groups were concentrated in metabolic pathways. Specifically, in the CK vs. TCS001 group, genes involved in glycine, serine, and threonine metabolism were significantly enriched. In the CK vs. VMA and TCS001 comparison, genes involved in oxidative phosphorylation, glycine, serine, and threonine metabolism, and cysteine and methionine metabolism were significantly enriched. A comparison between the CK vs. TCS001 group and the CK vs. VMA and TCS001 group revealed significant alterations in the enrichment of metabolic pathways following the combination treatment. This indicated that the treatments may exert a specific influence on the metabolic pathways of *Co. siamense.*

### 2.7. Effects of VMA and TCS001 Treatment on C. oleifera and Co. siamense

**Figure 6:**
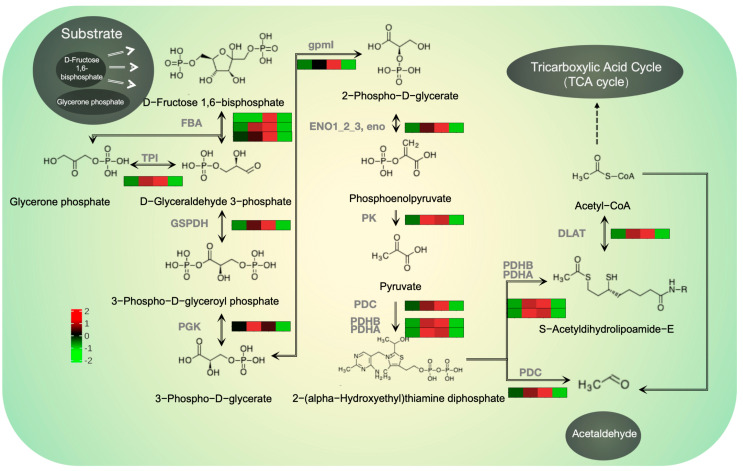
Hypothesis of the internal glycolytic/gluconeogenic pathway in *Colletotrichum siamense*. Each mini heatmap, from left to right, corresponds to the four sample groups (control group/VMA group, control group/TCS001 group, control group/VMA and TCS001 combined group, and control group alone). The expression terms within the heatmap represent the substrates within the pathway, with the names of the substrates listed below. The connected terms denote the enzymes regulated by the genes depicted in the heatmap.

Particular attention was paid to the glycolysis and gluconeogenesis pathways through the integration of the WGCNA with the enrichment analysis of DEGs. In this pathway, 23 DEGs were identified in the treatment group that received both VMA and TCS001 (17 upregulated and 6 downregulated). Notably, 13 of the upregulated genes occupied pivotal positions within the pathway and significantly influenced glycolysis and glucose biosynthesis in *Co. siamense*, which led to a substantial accumulation of acetaldehyde. The expression patterns of these 13 pivotal genes were comprehensively examined (Figure 6). The analysis revealed that the expression levels of these genes in the VMA-only treatment group (group A), the TCS001-only treatment group (group B), and the combined treatment group (group C) were all significantly higher than those in the control group (group CK). In particular, the amplitude of expression in the combined treatment group were markedly higher than those in the other groups, which indicated that the combined treatment of VMA and TCS001 had a significant impact on the glycolysis and glucose biosynthesis pathways of *Co. siamense*.

### 2.8. The Optimal Control Strategy of Combined Application of Chemicals

Three days after the last application, an investigation on the control effect on the leaves was conducted (Figure S1). Among the 15 treatments, the effect of the combined application of *B. velezensis* TCS001 and VMA was partially superior to the use of either agent alone. The best treatment was 100 mg/L + 5 × 10^6^ CFU/mL (Table 2), with a rate of 96.47%. Therefore, this treatment concentration was selected for the next in vivo test.

### 2.9. Effects of the Combination of VMA and B. velezensis TCS001 on the Disease Resistance of C. oleifera

**Figure 7:**
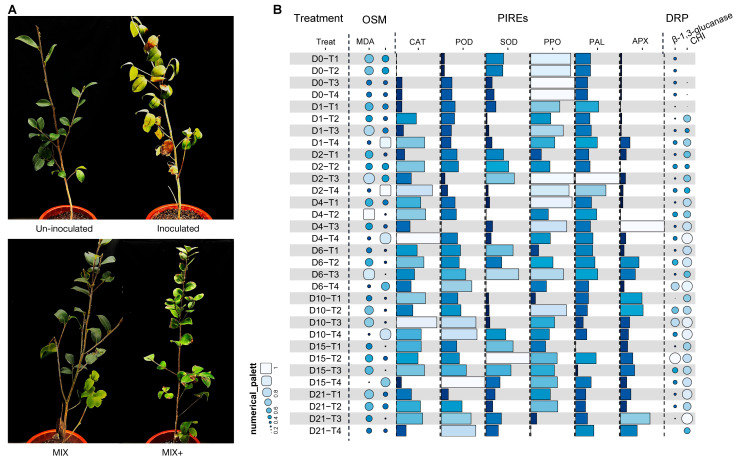
Evaluation of the potential of the co-application of validamycin A (VMA) and *Bacillus velezensis* TCS001 to activate disease resistance in *Camellia oleifera*, with associated physiological and biochemical indices. (**A**) Negative control was established by treating the samples with sterile water alone. The inoculated sample was treated with sterile water and subsequently inoculated with *Co. siamense*, which served as the positive control. The mixture was treated with VMA and TCS001 as a stand-alone treatment. MIX+: treated with VMA and TCS001 and inoculated with *Co. siamense*; (**B**) T1: un-inoculated; T2: inoculated; T3: MIX; T4: MIX+; OSM: oxidative stress marker; PIREs: plant immune response enzymes.

The un-inoculated group, which received only sterile water spray as a negative control, exhibited normal growth without any disease symptoms. The inoculated group, which served as the positive control, was sprayed with sterile water and inoculated with *Co. siamense*, which resulted in severe disease symptoms. This outcome was due to the immune response of *C. oleifera* to pathogen invasion, which induced numerous changes in disease resistance-related processes. The MIX group, treated exclusively with the VMA and TCS001 mixture, exhibited normal growth without evident disease symptoms. The MIX+ group was sprayed with the VMA and TCS001 mixture and inoculated with *Co. siamense*. Although this group exhibited disease symptoms, the affected area was smaller, and the damage was less severe compared with the inoculated group. Activity assays for MDA, O^2−^, and defense-related enzymes, including CAT, APX, SOD, POD, PAL, and PPO, as well as pathogenesis-related proteins such as β-1,3-glucanase (β-1,3-GLU) and CHI, were conducted on days 0, 1, 2, 4, 6, 10, 15, and 21 post-treatments across the four treatment groups. The results revealed the activation of related activities in the *C. oleifera* plants treated with the VMA and TCS001 mixture and provided insights into the mechanisms of disease resistance (Figure 7B).

Immediately after the application of treatments without pathogen inoculation, the levels of oxidative stress markers (OSMs) in the treated *C. oleifera* seedlings were significantly lower than those in the control group. Conversely, the levels of CAT, POD, and polyphenol oxidase (PPO) were markedly higher in the treated seedlings. This increase is attributable to the ability of the VMA and TCS001 mixture to enhance the defensive enzyme system of the plants, which reduced plant damage. On days 1 and 2 post-inoculation, both the inoculated and MIX + groups exhibited increased activities of defense-related proteins (DRPs) and pathogenesis-related enzymes (PIREs), particularly CAT and APX. Notably, in the MIX + group, the levels of CAT, SOD, PPO, PAL, and APX were significantly higher than those in the inoculated group, which served as the positive control.

Two days after pathogen inoculation, both the MIX and MIX+ groups exhibited a significant increase in PIRE content compared with the control group, with PPO and PAL predominantly enhanced. From day 4 to day 10, the levels of PIREs in the MIX+ group gradually decreased, which may be attributed to the completion of the immune response against the pathogen. From day 10 onward, the levels of DRP in plants treated with the VMA and TCS001 mixture decreased relative to the control group, which indicated an end to the course of the disease. By day 21, DRP levels stabilized compared with the control group, and this indicated the near completion of the disease-resistance process against the pathogen. Notably, the MDA content in the MIX+ group remained consistently low, while the O^2−^ content remained consistently high from the time of pathogen inoculation until day 15, after which it decreased and stabilized.

## 3. Discussion

*Bacillus*-based biocontrol agents (BCAs) have shown significant potential for the control of plant diseases [28,29]. However, current research mainly focuses on BCAs as alternatives to chemical fungicides, with limited investigation into the potential synergistic effects when co-administered with chemical agents. Most studies on the combined use involve mixing two BCAs or combining BCAs with fungicides, For example, Trichoderma bacteria co-inoculations have a synergistic effect on plant benefits [30],and combined application of *Bacillus subtilis* with the strobilurins showed synergistic effects [31]. Some studies have suggested that VMA enhances plant defense responses by stimulating the activity of defense-related enzymes within the plant [32,33]. Therefore, this study is the first to combine the disease-resistance potential of BCAs with the in vivo-induced resistance capability of VMA to activate immune responses in plants and investigate the underlying mechanisms. The findings of this study confirmed the significant antifungal and immune-activating properties of the combined VMA and TCS001 preparation, as observed in both in vitro and in vivo settings. Notably, in the in vitro tests, the mixed agents were VMA in its original form and the fermentation filtrate of TCS001, while in the pot experiments, the mixed agents were already marketed products.

In vitro experiments demonstrated that *Bacillus*-based agents possess the ability to inhibit a diverse range of fungal pathogens. For example, *B. velezensis* P2-1 has been shown to inhibit *Botryosphaeria dothidea* [34], while *B. subtilis* L1-21 has been shown to inhibit *Botrytis cinerea* [35]. Moreover, TCS001 in this study exhibited comparable inhibitory effects on *Co. siamense*. VMA has been shown to possess antibacterial properties, which inhibit the activity of trehalose [36,37,38] In this study, VMA exhibited inhibitory effects to an extent when used as a stand-alone agent. However, when VMA was co-administered with TCS001, the inhibitory effect was significantly greater than when used alone, which indicated a higher potential for development. Accordingly, the inhibitory mechanism of this mixed preparation was investigated, to provide a theoretical basis for future research into its potential applications. A combination of SEM and pathogenicity tests revealed that the mycelium exhibited a deformed morphology and lost its pathogenicity after treatment with VMA and TCS001. To gain further insight into the underlying molecular mechanisms of this phenomenon, transcriptomic data were subjected to a range of analytical techniques. This revealed, through WGCNA and KEGG enrichment analysis, that 13 key genes involved in the glycolysis/gluconeogenesis pathway of *Co. siamense* treated with the mixed preparation exhibited significant upregulation. This upregulation resulted in substantial acetaldehyde accumulation and apoptosis.

In vivo experiments were conducted to test the efficacy of the VMA and TCS001 mixed preparation. Oxidative stress in plant cells is induced by an increased leakage of electrons to O^2−^ during photosynthesis and respiration, and this enhances the generation of reactive oxygen species (ROS) [39]. ROS (O^2−^ and H_2_O_2_) production increases when plants are exposed to pathogens or other stressors [40]. The ROS can cause direct damage to cellular lipids, proteins, and DNA, which in turn leads to cell death. MDA serves as an indicator of lipid peroxidation [41]. The balance between ROS production and scavenging is closely regulated by plant antioxidant defense systems, and SOD, CAT, and POD are key PIREs that play a central role in ROS control [42,43]. DRPs are constitutively expressed at low levels in plants, but their expression is significantly upregulated upon infection by fungal, bacterial, or viral pathogens [44].

The most effective application strategy for activating the innate immune capabilities of *C. oleifera* seedlings was selected. The optimal in vivo concentration was identified, and measurements were taken for disease resistance indicators, as well as physiological and biochemical indicators. The results demonstrated that the immune response of the seedlings to anthracnose was prolonged and complex. This study validated the activation of disease resistance functions in *C. oleifera* seedlings treated with the VMA and TCS001 combination by observing the phenotype at eight time points over 21 days and measuring key disease resistance indicators such as MDA content, O^2−^ content, defense-related enzyme levels, and pathogenesis-related protein levels. These measurements revealed the physiological and biochemical mechanisms behind the disease resistance of the plant. In response to pathogens, plants typically increase the activity of defense-related enzymes to eliminate harmful substances and prevent further infection. However, research on the mechanisms of these defense enzyme changes is relatively limited. The findings of this study indicated that within 4–6 days after exposure to pathogens, the plant had largely undergone the necessary changes in the defense enzyme system. For example, CAT activity declined after 6 days in the inoculated group. This decline is attributable to the inability of the plant to produce defense enzymes, owing to functional impairment after pathogen invasion. The experiments revealed that in the MIX+ group, MDA content remained persistently low, while O^2−^ levels were persistently high from pathogen inoculation until day 15, after which it declined to a stable level. The underlying cause of this phenomenon is not entirely understood and requires further investigation.

In conclusion, the disease-resistance response of the plant followed a spirally ascending process. This process involved changes in specific disease resistance indicators and complex interactions between the plant and environmental factors (temperature, humidity, light, oxygen, and carbon dioxide concentration), as well as interactions between the different stages of pathogen infection and the internal response mechanisms of the plant. These findings lay the groundwork for further research on the disease resistance mechanisms of *C. oleifera* and their interactions. Additionally, they established a theoretical foundation for the application of VMA and TCS001 to activate the internal immune functions of *C. oleifera* against anthracnose disease. This research ultimately aims to ensure the high quality of tea oil and the safety of edible oil in China.

## 4. Materials and Methods

### 4.1. Test Fungi and Bacteria Culture Conditions

The strain *B. velezensis* TCS001 has been formally deposited with the China General Microbiological Culture Collection Center (CGMCC) and assigned the accession number CGMCC No. 8921.

The TCS001 strain was cultivated with a modified Luria–Bertani (MLB) medium formula, which comprised 7 g/L peptone (biosharp, Hefei, China), 2 g/L yeast extract (biosharp, Hefei, China), 6 g/L sodium chloride (biosharp, Hefei, China), 2 g/L glucose (macklin, Shanghai, China), 0.06 g/L potassium chloride (macklin, Shanghai, China), and 0.5 g/L magnesium chloride hexahydrate (macklin, Shanghai, China). The total volume of the medium was 1 L. The medium was prepared with sterile distilled water and adjusted to a pH of 7.1. Sterilization was conducted at 121 °C for 15 min. The strain culture was incubated at 145 rpm and 27 °C for 16 h before inoculation, at a 3% volume ratio, into the proprietary medium optimized for TCS001. This optimized medium contained 10.5 g/L soluble starch (sinopharm chemical reagent, Shanghai, China), 18.5 g/L peanut cake powder (sinopharm chemical reagent, Shanghai, China), and 3 g/L sodium chloride (biosharp, Hefei, China), with a working volume of 32% and a pH of 6.0, and it was fermented at 164 rpm and 31 °C for 48 h to yield the TCS001 fermentation broth. Solid cultures were cultivated on the Luria–Bertani (LB) agar medium, which comprised 18.0 g/L agar (sinopharm chemical reagent, Shanghai, China), 10.0 g/L peptone (biosharp, Hefei, China), 5.0 g/L yeast extract (biosharp, Hefei, China), and 10.0 g/L sodium chloride (biosharp, Hefei, China), with the pH adjusted to 7.0 and sterilized at 121 °C for 15 min.

*Co. siamense* was isolated from symptomatic *C. oleifera* leaf samples obtained from Zhejiang A&F University in Lin’an District, Hangzhou City, Zhejiang Province. The isolated pathogen was cultivated on potato dextrose agar (PDA) solid medium, prepared with 200 g/L of peeled potatoes, 20 g/L glucose (macklin, Shanghai, China), and 18 g/L agar (sinopharm chemical reagent, Shanghai, China), and then filled with distilled water to a total volume of 1 L. To prepare the conidial suspension, PDA plates with robust growth and no contamination were rinsed thrice with sterile water and then diluted to a concentration of 1 × 10^5^ conidia/mL using a hemocytometer (Thermo Scientific, Waltham, MA, USA) [45].

### 4.2. Evaluation of the Compatibility between VMA and TCS001

Sterile techniques were employed to transfer 1 mL of the TCS001 fermentation liquid, prepared according to the methods as detailed previously. A 100 μL aliquot of this liquid was uniformly spread across the surface of a sterile LB solid medium to represent the test microorganism. Disks of sterile filter paper (5 mm diameter) were immersed in concentrated VMA solution (2000 mg/L) for 5 s before being placed atop the LB solid medium inoculated with TCS001. Concurrently, sterile filter paper disks soaked in sterile water were used as a negative control. After inoculation, the plates were incubated in a culture chamber at 28 °C for 48 h. The emergence of a transparent zone of inhibition around the VMA-infused paper disks was monitored as an indicator of bacteriostasis. Conversely, the lack of such a zone would indicate compatibility between the two entities [46]. This procedure was conducted in triplicate to ensure consistency of the results.

### 4.3. Assessment of the In Vitro Inhibitory Activity against Co. siamense

The TCS001 fermentation broth was centrifuged at 7830 rpm and 4 °C for 30 min under aseptic conditions to separate the supernatant, known as the culture filtrate (CFS). The CFS was then filtered through a 0.22 μm membrane thrice to obtain the TCS001 fermentation filtrate. This filtrate was mixed with the PDA solid medium during the pre-gelation phase to create medicated culture media with drug-to-water ratios of 1:5, 1:10, 1:20, 1:40, and 1:80. Concurrently, 70% VMA technical material was prepared in the PDA medium at concentrations of 200, 100, 50, 25, and 12.5 mg/L, via the same procedure. Ten distinct concentrations, with five replicates each, were homogenized and transferred into 90 mm sterile Petri dishes. A Petri dish containing the PDA medium without any additives was used as the blank control.

After the aseptic techniques, mycelial blocks (8 mm diameter) were extracted from the PDA medium cultures containing *Co. siamense* hyphae. This method ensured the sterile collection of fungal biomasses for subsequent experimental analysis. The mycelial blocks were inoculated at the intersection of the cross on each medicated plate with a sterile inoculation needle. The inoculated plates were incubated at 25 °C for durations ranging from 72 to 96 h. When the colonies in the control plates occupied roughly three-quarters of the plate surface area, their diameters were measured via a cross-measurement technique. The mean of two such measurements was recorded as the colony diameter [47], and used to calculate the inhibition rate of mycelial growth [24]. The process was repeated thrice to ensure the consistency of the results.

The concentrations of VMA and the fermentation supernatant of TCS001 that exhibited the strongest antifungal activity was identified after an evaluation of the outcomes of the agar confrontation assay. These concentrations were then combined at an equal ratio (1:1) in the culture medium. The experiment was repeated to determine the synergistic impact of this mixture, and the inhibition rate (I) was calculated to quantify the overall inhibitory effect.

### 4.4. Changes in the Three-Dimensional Spatial Structure of Co. siamense Mycelium

The concentrations of VMA and fermentation supernatant of TCS001 with the strongest inhibitory effects were identified through the screening process as outlined previously. Subsequently, these concentrations were formulated into four discrete treatments: VMA, TCS001, a combination of VMA and TCS001 (hereafter referred to as VMA and TCS001), and a control (CK). Each treatment was replicated thrice. When the colonies on the control plates occupied roughly three-quarters of the surface area of the Petri dish, the solid medium containing the mycelium from the treated plates was sectioned into pieces measuring 0.5 cm × 0.3 cm. These pieces were then placed in 2 mL centrifuge tubes containing a 2.5% glutaraldehyde solution and maintained at 4 °C for 24 h for fixation. Subsequently, the mycelial pieces were rinsed thrice for 15 min, each with a 0.1 M, pH 7.0 PBS (Phosphate-Buffered Saline) buffer solution. The samples were then subjected to a graded ethanol series (30%, 50%, 70%, 80%, 90%, and 95%) for 15 min each for dehydration. After the ethanol was evaporated, the samples were washed twice with 100% ethanol for 20 min each. Subsequently, the samples were immersed in a mixture of ethanol and isopentyl acetate (1:1 *v*/*v*) for 30 min and then immersed in 100% isopentyl acetate for 2 h. The samples were adhered to a sample stage with a double-sided conductive adhesive, dried in a sterile environment, sputter-coated with gold, and finally examined under a scanning electron microscope to observe structural changes [48].

### 4.5. Assessment of the Pathogenic Potential of Mycelium with Varying Morphologies on C. oleifera

Mycelial disks (8 mm diameter) were aseptically extracted from the agar plates of fungi treated with VMA, TCS001, a combination of VMA and TCS001 (denoted as VMA and TCS001), and the control group (CK) using a sterile punch. Uniformly grown leaves of *C. oleifera* were selected and placed in sterile 90 mm culture dishes. The bottom of each dish was lined with a 90 mm sterile filter paper, and 5 mL of sterile reverse osmosis water was added to maintain a moist environment. The leaves were symmetrically wound on both sides of the midrib using a sterile needle. The sites of injury were inoculated with 40 μL of a 1% anhydrous glucose solution using a pipette. Subsequently, the prepared mycelial disks were placed over the sites of injury on the *C. oleifera* leaves. The inoculated leaves were cultivated under controlled conditions with a photoperiod of 16 h of darkness and 8 h of light, at 28 °C and 90% humidity. The progression of disease symptoms and the diameter of the lesions were monitored and recorded. The experiment employed four treatments, each replicated 20 times to ensure the reliability of the results [12].

### 4.6. Transcriptomic Analysis of Pathogens with Diverse Virulence Traits

Trizol Reagent (Solarbio, Beijing, China) was used to extract total RNA, after which a NanoDrop spectrophotometer (Thermo Scientific, MA, USA) was used to assess the concentration, quality, and integrity of the extracted samples. Furthermore, 3 µg of RNA was used as the input material for the RNA sample preparations. Sequencing libraries were generated according to the following steps. The first step involved the purification of mRNA from the total RNA using poly T oligo-attached magnetic beads. The fragmentation process was conducted using divalent cations under high temperatures in an Illumina-proprietary buffer. First-strand cDNA was synthesized using random oligonucleotides and SuperScript II reverse transcriptase. Subsequently, DNA polymerase I and RNase H were used to synthesize second-strand cDNA. The remaining overhangs were converted into blunt ends via exonuclease/polymerase activities, and the enzymes were removed. Illumina PE (Paired-End) adapter oligonucleotides were ligated in preparation for hybridization after adenylation of the 3′ ends of the DNA fragments. To select cDNA fragments of the preferred length (400–500 bp), the library fragments were purified using the AMPure XP system (Beckman Coulter, Beverly, CA, USA). The DNA fragments with ligated adapters on both ends were subjected to selective enrichment using the Illumina PCR (Polymerase Chain Reaction) Primer Cocktail in a 15-cycle PCR reaction. The products were then purified using the AMPure XP system and quantified using the Agilent high-sensitivity DNA assay on a Bioanalyzer 2100 system (Agilent, Santa Clara, CA, USA). Subsequently, the sequencing library was sequenced on a NovaSeq 6000 platform (Illumina, San Diego, CA, USA) by Shanghai Personal Biotechnology Co., Ltd. (Shanghai, China).

### 4.7. Integration of In Vivo and In Vitro Trials: Efficacy Screening for Disease Control

Seedlings of *C. oleifera* (*Changlin* 40 variety, 40–70 cm in height) were transplanted into pots (165 mm inner diameter and 150 mm height), each filled with a 1.2 kg mixture (1:1) of substrate soil and field soil. The pots had an internal volume of 1.2 L. The substrate soil was obtained from an agricultural supplier (Zhonghe agricultural, Huaian, China), while the field soil was obtained from the orchard at the Fruit Tree Garden of Zhejiang A&F University in Hangzhou. Subsequently, a layer of field soil was spread over the surface of the soil mixture. The trials were conducted in the greenhouse at the Green Pesticide 2011 Collaborative Innovation Center of Zhejiang A&F University, with conditions maintained at 26 ± 8 °C and 80% ± 10% humidity. From 20 October 2023 onward, pesticides were applied weekly, with a total of four spray applications conducted. Each application was administered at a rate of 40 mL per pot. The efficacy of the treatments was assessed 3 days after the final spray application using the detached leaf method, with five replicates per treatment. The treatment groups are presented in Table 3.

*C. oleifera* leaves of uniform size and age were immersed in a 1% sodium hypochlorite solution for 1 min, rinsed thrice with sterile water, and then left to air dry. The leaves were punctured on both sides with sterile needles, after which the leaves were inoculated with a conidial suspension of *Co. siamense* using a throat sprayer. After the leaves had dried, the petioles were wrapped in sterile, moistened cotton, and the leaves were placed in 90 mm Petri dishes lined with sterile filter paper. The control group consisted of leaves that had been inoculated with sterile water. The dishes were kept in the dark at a constant temperature of 28 °C and 95% relative humidity. A leaf area meter was used to measure the area of the lesions at the point of maximum disease symptom expression. The efficacy of the different treatments was evaluated based on the ratio of lesion area to total leaf area.

### 4.8. Study on the Enhancement of Disease Resistance in C. oleifera by Co-Application of VMA and TCS001

Two-year-old *C. oleifera* seedlings showing consistent growth were treated with a combination of VMA and TCS001. The treatment was administered bi-daily, with a total of three applications. An equivalent volume of sterile water was used as the control. On the second day following the final treatment, the seedlings were inoculated with a spore suspension of *Co. siamense*. The experimental groups were categorized as follows:

MIX+: Treated with VMA and TCS001 and inoculated with *Co. siamense*.

MIX: Treated with VMA and TCS001 alone.

Inoculated: Treated with sterile water and inoculated with *Co. siamense* (positive control).

Un-inoculated: Treated with sterile water alone (negative control).

Each treatment was replicated thrice, with a total of 10 seedlings per replicate. Leaves exhibiting uniform growth were sampled at 0, 1, 2, 4, 6, 10, 15, and 20 days post-inoculation, with each sample consisting of 10 leaves. The leaves were then flash frozen in liquid nitrogen for 15 min before storage at −80 °C for subsequent analysis [49]. According to the assay kit protocols, the levels of malondialdehyde (MDA) and superoxide anion (O^2−^) and the activity and rate of production of various defense-related enzymes were measured for the un-inoculated, inoculated, MIX, and MIX+ groups at 1, 2, 4, 6, 10, 15, and 20 days post-inoculation. The enzymes evaluated included catalase (CAT), ascorbate peroxidase (APX), superoxide dismutase (SOD), peroxidase (POD), phenylalanine ammonia-lyase (PAL), and polyphenol oxidase (PPO), along with pathogenesis-related proteins such as β-1,3-glucanase (β-1,3-GLU) and chitinase (CHI).

## 5. Conclusions

This study presents a promising strategy that combines low-toxicity chemical pesticides with microbial pesticides, specifically validamycin A (VMA) and *B. velezensis* TCS001, to control anthracnose in *C. oleifera*. The in vitro and in vivo experiments showed that this combination significantly enhanced the antifungal efficacy and activated the plant’s disease resistance response, leading to a robust immune response. Transcriptomic analysis revealed that the combined treatment disrupted the pathogen’s metabolism and redox processes, inducing apoptosis. This research not only provides valuable insights into the synergistic action of chemical and microbial pesticides but also paves the way for sustainable and effective disease management in forestry. The implementation of such integrated pest management strategies is essential to safeguard national edible oil production and ensure agricultural sustainability.

## Figures and Tables

**Table 1:** Statistical table of the number of differentially expressed genes.

Control_vs._Treat	Up	Down	Total
CK_vs._VMA	126	103	229
CK_vs._TCS001	1172	1049	2221
CK_vs._MIX	1310	878	2188
VMA_vs._TCS001	705	803	1508
VMA_vs._MIX	793	619	1412
TCS001_vs._MIX	359	125	484

**Table 2:** The optimal prevention and control strategy screening results of in vivo and in vitro combined experiments.

	Treatment	Concentration	Average Proportion of Disease Spots	Induction Resistance Effect
1	8% *Validamycin* Aq.Sol	200 mg/L	19.6%	62.24%
2		100 mg/L	2.4%	95.29%
3		50 mg/L	11.4%	77.98%
4		25 mg/L	33.9%	34.67%
5	2 × 10^9^ CFU/mL *Bacillus velezensis* TCS001 SC	1 × 10^7^ CFU/mL	10.2%	80.30%
6		5 × 10^6^ CFU/mL	9.7%	81.25%
7	8% *Validamycin* Aq.Sol + 2 × 10^9^ CFU/mL *Bacillus velezensis* TCS001 SC	100 mg/L + 1 × 10^7^ CFU/mL	10.6%	79.66%
8		100 mg/L + 5 × 10^6^ CFU/mL	1.8%	96.47%
9		50 mg/L + 1 × 10^7^ CFU/mL	14.6%	71.87%
10		50 mg/L + 5 × 10^6^ CFU/mL	2.5%	95.15%
11		25 mg/L + 1 × 10^7^ CFU/mL	4.2%	91.89%
12		25 mg/L + 5 × 10^6^ CFU/mL	2.9%	94.50%
13	50% *Polyoxins* W.P.	1000 mg/L	19.0%	63.31%
14	CK(Control Check) (inoculated)	/	51.9%	/
15	CK(Control Check) (un-inoculated)	/	/	/

**Table 3:** Processing of in vivo and in vitro bridging tests.

No.	Treatment	Concentration
1	8% *Validamycin* Aq.Sol	200 mg/L
2		100 mg/L
3		50 mg/L
4		25 mg/L
5	2 × 10^9^ CFU/mL *Bacillus velezensis* TCS001 SC	1 × 10^7^ CFU/mL
6		5 × 10^6^ CFU/mL
7	8% *Validamycin* Aq.Sol + 2 × 10^9^ CFU/mL *Bacillus velezensis* TCS001 SC	100 mg/L + 1 × 10^7^ CFU/mL
8		100 mg/L + 5 × 10^6^ CFU/mL
9		50 mg/L + 1 × 10^7^ CFU/mL
10		50 mg/L + 5 × 10^6^ CFU/mL
11		25 mg/L + 1 × 10^7^ CFU/mL
12		25 mg/L + 5 × 10^6^ CFU/mL
13	50% *Polyoxins* W.P.	1000 mg/L
14	CK(Control Check) (inoculated)	/
15	CK(Control Check) (un-inoculated)	/

The treatment agents used in the experiment were from Huifeng Technology Co., Ltd., Hangzhou, China.

## Data Availability

All data generated or analyzed during this study are included in this published article and its supplementary materials files.

## Supplementary Materials

The following supporting information can be downloaded at: https://www.mdpi.com/article/10.3390/plants13192743/s1, Figure S1: Processing of in vivo and in vitro bridging tests survey result; Table S1: Evaluation statistics of sequencing data.

## References

[1] Bai Y., Zhai Y., Ji C., Zhang T., Chen W., Shen X., Hong J. (2021). Environmental sustainability challenges of China’s edible vegetable oil industry: From farm to factory. Resour. Conserv. Recycl..

[2] Duan J., Nie C., Wang Y., Yan D., Xiong W. (2021). Research on global grain trade network pattern and its driving factors. Sustainability.

[3] Quan W., Wang A., Gao C., Li C. (2022). Applications of Chinese *Camellia oleifera* and its by-products: A review. Front. Chem..

[4] Gao L., Jin L., Liu Q., Zhao K., Lin L., Zheng J., Li C., Chen B., Shen Y. (2024). Recent advances in the extraction, composition analysis and bioactivity of *Camellia* (*Camellia oleifera* Abel.) oil. Trends Food Sci. Technol..

[5] Cao L., Sun X., Dong W., Ma L., Li H. (2023). Detection and quantification of anthracnose pathogen *Colletotrichum fructicola* in cultivated tea-oil *Camellia* species from southern China Using a DNA-Based qPCR assay. Plant Dis..

[6] Peng X.J., Wang Q.C., Zhang S.K., Guo K., Zhou X.D. (2023). Colletotrichum species associated with *Camellia anthracnose* in China. Mycosphere.

[7] Luan F., Zeng J., Yang Y., He X., Wang B., Gao Y., Zeng N. (2020). Recent advances in *Camellia oleifera* Abel: A review of nutritional constituents, biofunctional properties, and potential industrial applications. J. Funct. Foods.

[8] Gao X., Liu Y., Wang Q., Li B., Jiang X. (2021). Research of the content of nutrient elements caused by anthracnose to *Camellia oleifera* using LIBS technology. Appl. Phys. B.

[9] Lu Q., Wang Y., Li N., Ni D., Yang Y., Wang X. (2018). Differences in the characteristics and pathogenicity of *Colletotrichum camelliae* and *C. fructicola* isolated from the tea plant [Camellia sinensis (L.) O. Kuntze]. Front. Microbiol..

[10] Chen C., Chen H., Zhang Y., Thomas H.R., Frank M.H., He Y., Xia R. (2020). TBtools: An integrative toolkit developed for interactive analyses of big biological Data. Mol. Plant.

[11] Zhang S., Guo Y., Li S., Zhou G., Liu J., Xu J., Li H. (2019). Functional analysis of *CfSnf1* in the development and pathogenicity of anthracnose fungus *Colletotrichum fructicola* on tea-oil tree. BMC Genet..

[12] Yang C., Wu P., Yao X., Sheng Y., Zhang C., Lin P., Wang K. (2022). Integrated transcriptome and metabolome analysis reveals key metabolites involved in *Camellia oleifera* defense against *Anthracnose*. Int. J. Mol. Sci..

[13] Meng J., Zhang X., Han X., Fan B. (2022). Application and development of biocontrol agents in China. Pathogens.

[14] Ye H.-L., Chen Z.-G., Jia T.-T., Su Q.-W., Su S.-C. (2021). Response of different organic mulch treatments on yield and quality of *Camellia oleifera*. Agric. Water Manag..

[15] Zhang P., Cui Z., Guo M., Xi R. (2020). Characteristics of the soil microbial community in the forestland of *Camellia oleifera*. PeerJ.

[16] Cui Y., Xu Z., Tang S., Wang Y., Jiang G. (2022). Organochlorine pesticides and other pesticides in peanut oil: Residue level, source, household processing factor and risk assessment. J. Hazard. Mater..

[17] Rajput S., Sharma R., Kumari A., Kaur R., Sharma G., Arora S., Kaur R. (2021). Pesticide residues in various environmental and biological matrices: Distribution, extraction, and analytical procedures. Environ. Dev. Sustain..

[18] Chen X., He Y., Wang Z., Niu A., Xue Y., Zhou D., Zhou G., Liu J. (2023). Research progress and management strategies of fungal diseases in *Camellia oleifera*. Front. Microbiol..

[19] Lahlali R., Ezrari S., Radouane N., Kenfaoui J., Esmaeel Q., El Hamss H., Belabess Z., Barka E.A. (2022). Biological control of plant pathogens: A global perspective. Microorganisms.

[20] El-Baky N.A., Amara A. (2021). Recent Approaches towards control of fungal diseases in plants: An Updated Review. J. Fungi.

[21] Pandit M.A., Kumar J., Gulati S., Bhandari N., Mehta P., Katyal R., Rawat C.D., Mishra V., Kaur J. (2022). Major biological control strategies for plant pathogens. Pathogens.

[22] Khan A.R., Mustafa A., Hyder S., Valipour M., Rizvi Z.F., Gondal A.S., Yousuf Z., Iqbal R., Daraz U. (2022). *Bacillus* spp. as bioagents: Uses and application for sustainable agriculture. Biology.

[23] Salwan R., Sharma M., Sharma A., Sharma V. (2023). Insights into plant beneficial microorganism-triggered induced systemic resistance. Plant Stress.

[24] Jin J., Yang R.D., Cao H., Song G.N., Cui F., Zhou S., Yuan J., Qi H., Wang J.D., Chen J. (2024). Microscopic and Transcriptomic Analyses to elucidate antifungal mechanisms of *Bacillus velezensis* TCS001 Lipopeptides against *Botrytis cinerea*. J. Agric. Food Chem..

[25] Zehra A., Raytekar N.A., Meena M., Swapnil P. (2021). Efficiency of microbial bio-agents as elicitors in plant defense mechanism under biotic stress: A review. Curr. Res. Microb. Sci..

[26] Bian C., Duan Y., Wang J., Xiu Q., Wang J., Hou Y., Song X., Zhou M. (2020). Validamycin A Induces Broad-Spectrum Resistance Involving Salicylic Acid and Jasmonic Acid/Ethylene Signaling Pathways. Mol. Plant Microbe Interact..

[27] Ons L., Bylemans D., Thevissen K., Cammue B.P.A. (2020). Combining biocontrol agents with Cchemical Fungicides for integrated plant fungal disease control. Microorganisms.

[28] Heo Y., Lee Y., Balaraju K., Jeon Y. (2023). Characterization and evaluation of *Bacillus subtilis* GYUN-2311 as a biocontrol agent against *Colletotrichum* spp. on apple and hot pepper in Korea. Front. Microbiol..

[29] Maral-Gul D., Eltem R. (2024). Evaluation of *Bacillus* isolates as a biological control agents against soilborne phytopathogenic fungi. Int. Microbiol..

[30] Poveda J., Eugui D. (2022). Combined use of Trichoderma and beneficial bacteria (mainly *Bacillus* and *Pseudomonas*): Development of microbial synergistic bio-inoculants in sustainable agriculture. Biological Control.

[31] Liu L., Liang M., Li L., Sun L., Xu Y., Gao J., Wang L., Hou Y., Huang S. (2018). Synergistic effects of the combined application of Bacillus subtilis H158 and strobilurins for rice sheath blight control. Biol. Control.

[32] Naz M., Zhang D., Liao K., Chen X., Ahmed N., Zhou J.-J., Chen Z. (2024). The Past, Present and Future of Plant Activator Targeting Salicylic Acid Signal Pathway. Portico.

[33] Yan F., Ma J., Peng M., Xi C., Chang S., Yang Y., Tian S., Zhou B., Liu T. (2024). Lactic acid induced defense responses in tobacco against *Phytophthora nicotianae*. Sci. Rep..

[34] Yuan H., Shi B., Wang L., Huang T., Zhou Z., Hou H., Tu H. (2021). Isolation and characterization of *Bacillus velezensis* strain P2-1 for biocontrol of apple postharvest decay caused by *botryosphaeria dothidea*. Front. Microbiol..

[35] Bu S., Munir S., He P., Li Y., Wu Y., Li X., Kong B., He P., He Y. (2021). *Bacillus subtilis* L1-21 as a biocontrol agent for postharvest gray mold of tomato caused by *Botrytis cinerea*. Biol. Control.

[36] Zhao B., Li J., Zhou L., Liu W., Geng S., Zhao Y., Hou Z., Zhao R., Liu Y., Dong J. (2024). Validamycin A inhibited FB(1) biosynthesis by the target *FvNth* in *Fusarium verticillioides*. J. Agric. Food Chem..

[37] Lu Y., Ye K., Zhu L., Cai X., Yang S., Li J., Jiang R., Fan Y., Chen X. (2021). Synthesis of a series of validoxylamine A esters and their biological activities. Pest Manag. Sci..

[38] Bian C., Duan Y., Xiu Q., Wang J., Tao X., Zhou M. (2021). Mechanism of validamycin A inhibiting DON biosynthesis and synergizing with DMI fungicides against *Fusarium graminearum*. Mol. Plant Pathol..

[39] Chaki M., Begara-Morales J.C., Barroso J.B. (2020). Oxidative stress in plants. Antioxidants.

[40] Perez-Torres I., Castrejon-Tellez V., Soto M.E., Rubio-Ruiz M.E., Manzano-Pech L., Guarner-Lans V. (2021). Oxidative stress, plant natural antioxidants, and obesity. Int. J. Mol. Sci..

[41] Cordiano R., Di Gioacchino M., Mangifesta R., Panzera C., Gangemi S., Minciullo P.L. (2023). Malondialdehyde as a potential oxidative stress marker for allergy-oriented diseases: An update. Molecules.

[42] Jin X., Liu Z., Wu W. (2022). POD, CAT and SOD enzyme activity of corn kernels as affected by low plasma pretreatment. Int. J. Food Prop..

[43] Che Y., Zhang N., Zhu X., Li S., Wang S., Si H. (2020). Enhanced tolerance of the transgenic potato plants overexpressing Cu/Zn superoxide dismutase to low temperature. Sci. Hortic..

[44] Liu H., Lu X., Li M., Lun Z., Yan X., Yin C., Yuan G., Wang X., Liu N., Liu D. (2023). Plant immunity suppression by an exo-beta-1,3-glucanase and an elongation factor 1alpha of the rice blast fungus. Nat. Commun..

[45] Choi Y.-W., Hyde K.D., Ho W. (1999). Single spore isolation of fungi. Fungal Divers..

[46] Zhe C., Jing H., Jia Z., Hong L. (2018). Screening of the combinations of *Bacillus* Strains against strawberry *Anthracnose*. Chin. J. Biol. Control.

[47] Yu X., Li J., Mu D., Zhang H., Liu Q., Chen G. (2021). Green synthesis and characterizations of silver nanoparticles with enhanced antibacterial properties by secondary metabolites of *Bacillus subtilis* (SDUM301120). Green. Chem. Lett. Rev..

[48] Baptista J.P., Teixeira G.M., de Jesus M.L.A., Berte R., Higashi A., Mosela M., da Silva D.V., de Oliveira J.P., Sanches D.S., Brancher J.D. (2022). Antifungal activity and genomic characterization of the biocontrol agent *Bacillus velezensis* CMRP 4489. Sci. Rep..

[49] Arias Padro M.D., Caboni E., Salazar Morin K.A., Meraz Mercado M.A., Olalde-Portugal V. (2021). Effect of *Bacillus subtilis* on antioxidant enzyme activities in tomato grafting. PeerJ.

