# Supplementary material for: Enhanced Antifungal Efficacy of Validamycin A Co-Administered with Bacillus velezensis TCS001 against Camellia anthracnose
Source: Plants (Basel). 2024 Sep 30;13(19):2743. doi: 10.3390/plants13192743 (PMC11479143; doi:10.3390/plants13192743)
Supplement: Supplementary file 1 [file plants-13-02743-s001.zip › plants-3214963-supplementary.pdf]

Supplementary Information

Enhanced Antifungal Efficacy of Validamycin A Co-Administered with *Bacillus velezensis* TCS001 against *Camellia anthracnose*

Zhilei Chen [1], Hao Cao [1], Jing Jin [1], Zhong Li [2], Shouke Zhang [1,*] and Jie Chen [1,*]

The following Supporting Information is available for this article:

Figures S1

Tables S1

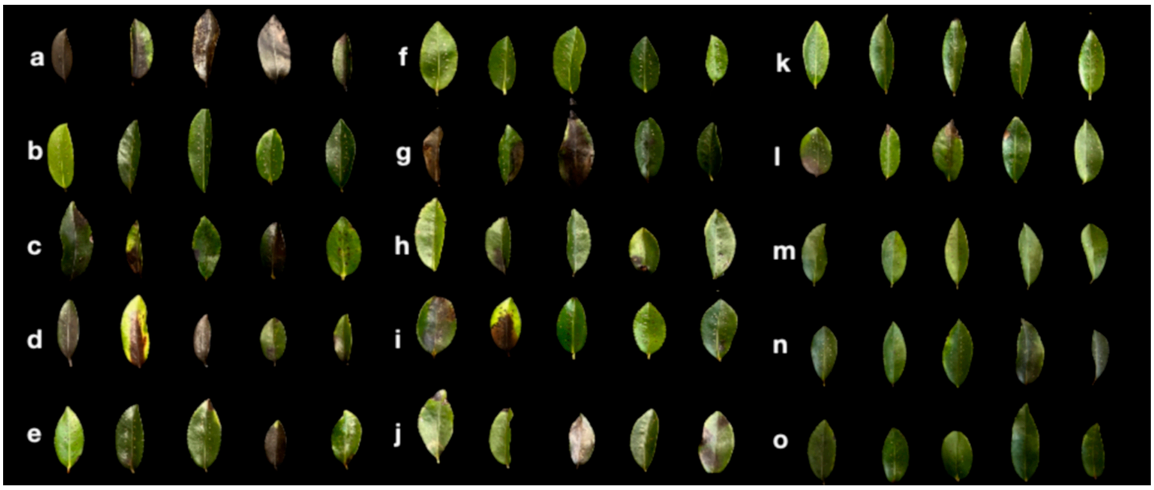

Figure S1. Processing of in vivo and in vitro bridging tests survey result.

(a-o) CK (inoculated); CK (Un-inoculated); 50% *Polyoxins* W.P. 1000mg/L; 8% *Validamycin* Aq.Sol 25mg/L; 8% *Validamycin* Aq.Sol 50mg/L; 8% *Validamycin* Aq.Sol 100mg/L;8% *Validamycin* Aq.Sol 200mg/L; 2×10^9CFU/mL *Bacillus velezensis* TCS001 SC 5×10^6CFU/mL;2×10^9CFU/mL *Bacillus velezensis* TCS001 SC 1×10^6CFU/mL; 8% *Validamycin* Aq.Sol+2×10^9CFU/mL *Bacillus velezensis* TCS001 SC=100mg/L+5×10^6CFU/mL;8% *Validamycin* Aq.Sol+2×109CFU/mL *Bacillus velezensis* TCS001 SC=50mg/L+1×10^7CFU/mL;8% *Validamycin* Aq.Sol+2×10^9CFU/mL *Bacillus velezensis* TCS001 SC=50mg/L+5×10^6CFU/mL;8% *Validamycin* Aq.Sol+2×10^9CFU/mL *Bacillus velezensis* TCS001 SC=25mg/L+1×10^7CFU/mL;8% *Validamycin* Aq.Sol+2×10^9CFU/mL *Bacillus velezensis* TCS001 SC=25mg/L+5×10^6CFU/mL.

Table S1. Evaluation statistics of sequencing data

Sample	Raw reads	Clean reads	Clean bases	Useful reads
A1	38143606	37623852	5676001587	98.64%
A2	42230560	41733490	6296245206	98.82%
A3	40313624	39673610	5985435814	98.41%
A4	47858720	47230640	7124636320	98.69%
B1	42590066	41999724	6335137434	98.61%
B2	40667070	40112614	6048529231	98.64%
B3	43102864	42454730	6399885177	98.50%
B4	41704260	41221156	6214367358	98.84%
C1	41284646	40766438	6146284791	98.74%
C2	38624630	38137578	5749567116	98.74%
C3	52880670	52301274	7887525122	98.90%
C4	37153280	36673586	5532455409	98.71%
CK1	46454684	45897526	6921011641	98.80%
CK2	46556068	45979052	6933023571	98.76%
CK3	47293178	46703244	7046068144	98.75%
CK4	37383650	36867896	5562921217	98.62%

Sample	Uniquely mapped	Multiple mapped	Q30 (%)	Raw N rate
A1	286166 (0.88%)	32286540 (99.12%)	96.59	0.009375
A2	210253 (0.58%)	35753561 (99.42%)	96.8	0.009456
A3	258029 (0.75%)	34187159 (99.25%)	95.88	0.008707
A4	292904 (0.72%)	40503116 (99.28%)	96.65	0.009402
B1	259727 (0.72%)	35773177 (99.28%)	96.34	0.008812
B2	230579 (0.66%)	34704518 (99.34%)	96.59	0.009454
B3	217253 (0.60%)	35995306 (99.40%)	95.97	0.008553
B4	211334 (0.59%)	35322895 (99.41%)	96.85	0.009334
C1	179069 (0.52%)	33977622 (99.48%)	96.76	0.009369
C2	205584 (0.63%)	32371619 (99.37%)	96.7	0.00941
C3	318896 (0.69%)	46102268 (99.31%)	96.91	0.009399
CK1	339027 (0.85%)	39376416 (99.15%)	96.87	0.009379
CK2	278462 (0.70%)	39495803 (99.30%)	96.74	0.009486
CK3	399672 (0.99%)	39837894 (99.01%)	96.78	0.009483
CK4	280781 (0.88%)	31711173 (99.12%)	96.47	0.009556
